# Evidence-based Healthcare and Health Informatics: Derivations and Extension of Epidemiology

**DOI:** 10.2188/jea.16.93

**Published:** 2006-05-19

**Authors:** Takeo Nakayama

**Affiliations:** 1 Department of Health Informatics, Kyoto University School of Public Health.

**Keywords:** Information Management, Evidence-Based Medicine, Communication, accountability, Decision Making

## Abstract

Epidemiology provides extremely valid information and evidence regarding human health. Epidemiologic findings with regard to major illnesses must be amassed, enhanced, and expanded further into related areas as a foundation for evidence-based medicine that is based on clinical practice, as well as for evidence-based healthcare that includes public health-related issues. Epidemiology should be recognized not only by epidemiologists but also by a variety of people, including specialists in other areas for healthcare and medicine, people in law and media, policy makers, and the general public. A system is needed that can create information for facilitating appropriate decision-making with issues related to clinical medicine and public health. The principles and methodology of epidemiology are used as a base for developing a field of health informatics. The objective of health informatics is to establish a system for facilitating the flow and circulation of health and medical information. Health informatics has potential applications for the creation, communication, and use of information, and the discipline is being expanded as a practical applied science in search of solutions. This report represents an effort to expand the scope of health informatics and extend the applications of epidemiology by working with individuals in other disciplines and the public.

The word “information,” which is very common, is defined in *The American Heritage Dictionary* as “the act of informing or condition of being informed; communication or reception of knowledge; knowledge derived from study, experience, or instruction; facts.” In addition, Claude Shannon, a mathematician and developer of digital theory, defined information as “that which decreases uncertainty”^1^ in decision-making, and Gregory Bateson, a cultural anthropologist, described it as “any difference that makes a difference.”^2^ The objective of health informatics is to provide information that will empower people to solve problems by applying health informatics in combination with evidence-based healthcare that comes from epidemiologic research.

## Epidemiologic Research: Promotion of Collaborative Studies and the Development of a System for Research Ethics Review

In the beginning of research career, the author studied the risk factors of cardiovascular disease using cohort studies based on community.^3^^-^^5^ Relationships have been noted between physical inactivity and cerebral hemorrhage in women and heavy labor and cerebral infarction as well as smoking and all types of stroke in men. As a result of participating in an international prospective collaborative study, contributions were made toward the consolidation of epidemiologic information on cardiovascular disease.^6^^,^^7^ In addition, by focusing on the attributable risk as an index to link epidemiologic causality with policymaking significance,^8^^,^^9^ the attributable fraction of stroke incidence in middle age people was reported to be 15% with smoking, 4% with uncontrolled hypertension, and 14% with untreated hypertension.^10^

In collaborative studies with clinicians, the biochemical marker Ki-67 was identified for use in the prognosis of cerebral carcinoma independently of pathological staging.^11^ In another study, spacers were observed to protect against mandibular complications in low-dose brachytherapy for oral tongue carcinoma.^12^ An epidemiologic study was also conducted on the quality of life (QOL) in patients with severe myopia^13^ and on the relationship between labor stress and depression/suicide.^14^^,^^15^ The latter report, a collaboration with psychiatrists on a case series of suicide attributed to overwork^15^ was the first detailed descriptive study of its type regarding this phenomenon, which is unique to Japan. Currently, the author is leading a multi-center collaborative study on QOL following acute myocardial infarction under a grant-in-aid by the Ministry of Education, Culture, Sports, Science and Technology of Japan.

Prior to the increase in concerns about research ethical guidelines, the author assessed the degree of understanding on research participation with a survey intended for the general population.^16^ The author was also involved in a working group that was supported by a grant-in-aid from the Ministry of Health and Welfare (MHW) (currently, the Ministry of Health, Labour and Welfare (MHLW); principal investigator: Akiko Tamakoshi) to investigate the ethics of epidemiologic research. The same working group drafted guidelines governing the informed consent of study participants in epidemiologic research in 2000.^17^^,^^18^ At the same time, under the guidance of the author as a principal investigator, the MHW working group was assessing epidemiologic research to determine any potential contributions to health policy. This group proposed the future modality of epidemiologic research and the verification of its meaning from the perspective of social accountability (Table 1).^19^

**Table 1.** Epidemiology in the 21st Century: A proposal of requirements

1	To comply with relevant laws and guidelines, including personal information protection
2	To conduct research in close collaboration with society
3	To utilize information adequately
4	To provide results that meet the expectation of society and foster trust
5	To responsibly conduct epidemiology research
6	To present findings to the public

These requisites were proposed by a working group of the Ministry of Health and Welfare; their project is titled “Assessment of Epidemiologic Research in Terms of Contributions to Health Policy.” (April 2000) (in Japanese)

At the present time, the author is a member of the institutional review boards of the following organizations: the Japan Epidemiological Association (the western Japan division), the Translational Research Institute of the Foundation for Biomedical Research and Innovation, the NPO (non-profit organization) Institute for Health Outcomes & Process Evaluation Research, and the Subcommittee of Epidemiologic and Clinical Research, Institutional Review Board of Kyoto University School of Medicine.

## Toward Evidence-based Medicine and Healthcare

**Figure 1.**
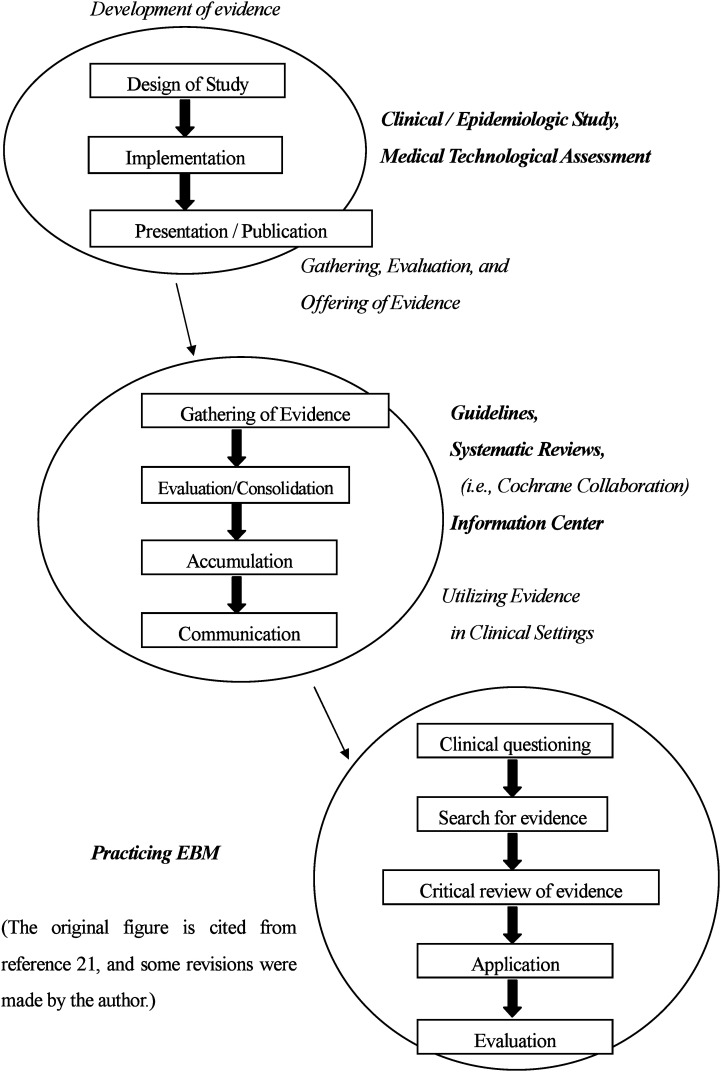
The Process of Evidence-based Medicine (EBM).

The concept of evidence-based medicine (EBM) was developed and proposed by Guyatt^20^ in 1991 and has been used in clinical medicine and by public health organizations as a result of the revolutionary advances in information technology such as the Internet and databases (Figure 1).^21^ Furthermore, Gray^22^ advocated the use of evidence-based healthcare as an expansion of EBM by noting that the science that is the most relevant to healthcare decision-making is epidemiology, which is the study of disease in groups of patients and populations.

To renew and integrate information in healthcare matters, guidelines play an important role in clinical practice and in public health settings. The US Institute of Medicine states that “clinical practice guidelines are systematically developed statements to assist practitioner and patient decisions about appropriate health care for specific clinical circumstances.” Related to the ever-increasing interest in “evidence-based clinical practice guidelines” in Japan, which initially began with those developed by the MHLW,^23^^,^^24^ epidemiologists who are knowledgeable about research design and information quality are eagerly expected to participate in the developmental process.^25^^,^^26^ The author has been involved in several projects in which guidelines were developed. They include an MHLW research group for rheumatoid arthritis,^27^ focal treatment of pressure ulcers (the Japanese Society of Pressure Ulcers),^28^ acute otitis media (Otological Society of Japan), management of dysphagia (the Oto-rhino-laryngological Society of Japan), smoking cessation (MHLW Research Project), and cancer screening (MHLW Cancer Research Project).^29^ The author has also examined the guidelines for domestic violence during the perinatal period (St Luke’s University Project of the Center of Excellence)^30^ as an external reviewer. Currently, the author is working collaboratively to evaluate the influence of clinical practice guidelines with the Japan Rheumatism Foundation, the Japanese Society of Neurology, the Japanese Orthopedic Association, and the Liver Cancer Study Group of Japan.

The author has also been working as the principal investigator of the following MHLW working groups in the field of EBM and practice guidelines: “A study on the acceptability and developmental methodology of ‘structured abstracts’ to be used for medical databases and EBM-oriented ‘Clinical Practice Guidelines (2001-2003)’”^31^^,^^32^ and “A study on the infrastructure development for the appropriate development, use, and distribution of ‘Evidence-based Guidelines (2004-).’” Much attention has been given to the role of clinical practice guidelines as a tool to promote communication and share information among patients, care givers, and health professionals, including clinicians. Although patient participation in the development stage of clinical practice guidelines is necessary to achieve this purpose, these attempts have been very rare in Japan. The author assisted the NPO “Allergypot,” a support organization for asthmatic and allergic children, which was collaborating with the MHLW working group in the development of clinical practice guidelines for asthma. As a result, efforts to formulate clinical practice guidelines for the general public regarding the treatment of asthma were successful.

The development of evidence-based clinical practice guidelines requires more than works within a professional society, which review clinical evidence scientifically and make recommendations in terms of clinicians. The sharing and integration of information that comes from a consensus among stakeholders including patients and a transparent process is indispensable. It is necessary to improve these methodologies. Furthermore, collaboration among medical professionals and lay people is expected to contribute to shared knowledge and have an influence on medical resources and user demands.^34^ Individuals employed by mass media are invited to participate in official meetings of some groups working on our project on evidence-based clinical practice guidelines and to make presentations in the course of open forums. It is important to exchange ideas and share experiences between health professions and individuals with mass media. The project described in this paper has been sometimes covered by the mass media (Table 2).

**Table 2.** Mass media reporting of the project on evidence-based clinical practice guidelines (2003-5).

1	“Standard treatment, easy to understand for patients,” Nihon Keizai Shimbun (April 28, 2003)
2	“Patient participation in developing clinical practice guidelines for the treatment of asthma,” Asahi Shimbun (June 13, 2004)”
3	“Q & A: What are clinical practice guidelines?” Yomiuri Shimbun (January 25, 2005)
4	“EBM clinical practice guidelines,” Yomiuri Shimbun (September 21, 2005)

The maintenance of a database is essential, and, as a working and committee member, the author has contributed to the improvement of domestic databases for medical information. First, the Japan Council for Quality Health Care launched the Medical Information Network Distribution Service (*Minds*) in 2002.^35^ Minds was designed to serve as a clearinghouse of clinical practice guidelines developed in Japan. It provides users with useful clinical information from recent articles, and its contents are accessible to anyone, including laypeople. Second, the Igaku-Chuo-Zasshi (*Ichushi*) Web provided by the Japan Medical Abstract Society (Japana Centra Revuo Medicina), the most longstanding domestic database for medical literature, began a new tagging system in terms of research design and clinical practice guidelines in 2003.

The abuse and misuse of impact factors in the evaluation of academic information has become an international problem. Impact factors include mere citations of reports published in journals without a qualitative evaluation of each quotation. On the other hand, evidence-based clinical practice guidelines identify important clinical and public health problems and review articles after having searched and evaluated to determine the usefulness to decision-making. Accordingly, journals and academic information can be evaluated by using a citation analysis of evidence-based guidelines, and the results of such an evaluation would differ from those obtained from impact factors and common citation analyses. A citation analysis of recent guidelines accessible online via the United States *Guide to Clinical Preventive Services (3rd Edition)* produced only a weak correlation between the number of quotations in the guidelines and the ranking by impact factor for academic journals.^32^ The contribution to problem solving and decision making in terms of EBM was found to be different from that obtained in a conventional evaluation using impact factors in preventive medicine and epidemiologic research (or journals). To promote clinical or epidemiologic studies that are relevant for decision-making, an alternative system for evaluating the academic output in these fields needs to be established. Within the MHLW working group on clinical practice guidelines noted above, the author began a project in 2005 with *Thomson Scientific*, which compiles and issues the science citation index and the impact factors, to examine which articles and which journals are cited in evidence-based clinical practice guidelines in each subject. The results will provide a new perspective on citation analysis and, furthermore, an assessment of the productivity of academic and clinical investigators or epidemiologists in terms of evidence-based concepts.

## Developing a New Field: Health Informatics

Health informatics is a new field that seeks to verify the quality of information related to health and medicine and promote communication among stakeholders. The use and impact of health informatics on behavior and health outcomes and the modality of information used to support human health, health behavior, decision-making, and problem-solving are challenging subjects to be investigated. This approach is based on clinical medicine, public health, informatics, and behavioral sciences, and, especially, on epidemiology and EBM. Health informatics is derived from epidemiology and has potential as a new academic discipline for the promotion of evidence-based healthcare.

**Figure 2.**
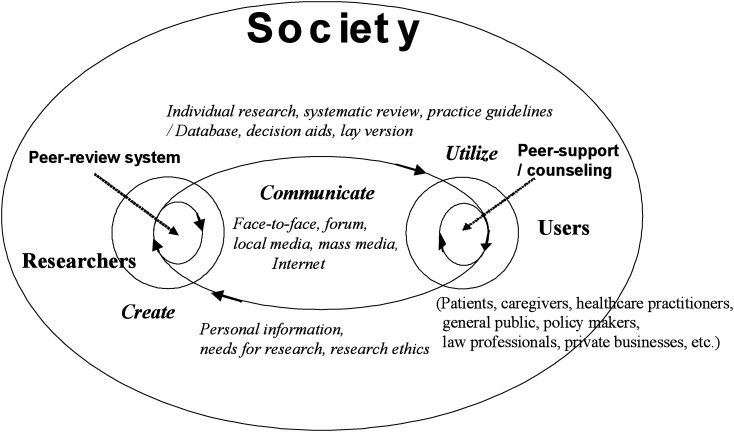
Flow and circulation of health and medical information in society.

Figure 2 describes the concept of expanding the idea of EBM to the flow and circulation of medical and health information in society. It is primarily the researcher who develops information related to health and medical subjects. Users of health and medical information include, but are not limited to, healthcare practitioners, patients and caregivers, the general public, policy-makers, and private individuals in business and law. Generally, researchers do not present their findings with the intention that they will be read by people outside of their own specialist community. Researchers primarily focus on peer review in academic journals, a system whereby colleagues from the same field assess the results of the presenter’s research.

Information that is intended for peer review would be difficult for laypeople to understand. Research is currently communicated by face-to-face communication, the local media, open lectures, and forums, as well as by the mass media, including research written for laypeople, and the Internet. Some advanced research groups have appointed publicists to their research projects.^36^ Appropriate communication of research findings and methodology to the general public requires accurate reporting.

In addition to the efforts made by individual researchers to communicate their own research, it is necessary to develop human resources that can transmit information accurately between researchers and the general public in Japan. Such professionals are called “medical writers” or “health communicators.” Epidemiologists are sometimes responsible for the training of professional communicators. At a symposium sponsored by the Japan Medical and Scientific Communicators Association (JMCA) in November 2004, the author gave the keynote lecture on the problems of medical information and medical communicators in health communications.^37^ The author is currently working to enhance the value of health informatics by collaborating with professional managers of health communications and by establishing relationships with mass media communicators.

In Japan, researchers are generally passive with regard to the mass media, and, as a result, the quality of information in the mass media is largely dependent on the knowledge of the journalist or reporter. Therefore, it would be valuable to assess the quality of news reports of research findings published in the mass media from the viewpoint of researchers. For example, “The Media Doctor,” a program introduced in 2004 at New Castle University, Australia, assesses the quality of newspaper articles on medical technology.^38^ Such an attempt may facilitate the appropriate flow of valid information in the mass media. Epidemiologists can watch the quality of information related health or disease risk released in mass media and make some proposals to improve them.

The next step for public health researchers is that active transmission of professional messages to the general public through the mass media, a process that is known as “media advocacy.” This approach is gaining significant interest in Europe and the United States.^39^ The Media Advocacy Manual developed by the American Public Health Association (APHA) states the following: “Advocacy is used to promote an issue in order to influence policy makers and encourage social change. Advocacy in public health plays a role in educating the public, swaying public opinion or influencing policy makers.”^40^ It may be the time to consider the importance and possibilities of such an attempt in Japan. Any advocacy should only be based on sound evidence.

Integrating and processing original information is an important part of communicating information effectively. This process includes clinical practice guidelines, systematic reviews, and furthermore, a decision aid or decision support tool.^41^ One of the studies undertaken by the MHLW working group is U-CARE (Unruptured Cerebral Aneurysm study for better Risk communication and Evidence-based decision making). The author is now promoting a collaborative project to develop an interactive decision aid for people who have been diagnosed with unruptured cerebral aneurysms.^42^ This project, called U-SHARE (Ubiquitously Support and Heal, Aneurysmal patients with Risk Communication and Empowerment), comprises the data of large-scale domestic and foreign epidemiologic studies, qualitative analysis of patient needs and concerns by semi-structured interview and decision analysis obtained with the use of the Markov model, and an interactive interface obtained through information technology.

The Japanese government reported that 63% of the general population of Japan was using the Internet at the end of 2004.^43^ Several problems are associated with placing information on the Internet. Information can be easily posted and accessed on the Internet, which makes the quality of such information questionable. The abundance of information available on the Internet is sometimes an impediment to accurate decision-making. Therefore, tools for rating quality of health information on the internet are required.^44^ In the United States, the American Medical Association (AMA) proposed guidelines for medical and health information sites on the Internet in 2000.^45^ The AMA guidelines served as an inspiration to develop an e-Health Ethics Code to be published by the NPO Japan Internet Medical Association (JIMA: http://www.jima.or.jp/), in which the author has been involved. This code covers content, communication, care, commerce, and privacy, and the objective is to support Website developers who are responsible for posting and receiving information on the Internet. This project provides insight into how to evaluate the quality of Internet information. As of November 2005, 13 hospitals and clinics had been authorized to participate in this project.

Peer counseling and support for information users are receiving more interest from health professionals and the general public. Internet developments, such as mailing lists and message boards, are having a positive impact on this movement. Furthermore, social networking sites (SNS), which are community Websites that begin with individuals with common interests, are expanding rapidly. Any information disseminated through these Internet functions may be beyond the control of any authority; as a result, the information could be biased or plainly wrong. Healthcare research has the objective of disseminating accurate information and evidence to aid individuals make proper decisions with regard to their health.

Nowadays, ethics and scientific integrity are extremely important relative to research. Ethical guidelines governing research and the protection of privacy are applicable to epidemiologists and all research scientists in the medical and healthcare professions. Researchers are also accountable for their research to society, e.g., for explaining the purposes, methods, and importance of their research to lay people in an understandable manner. In Japan, after the Act on the Protection of Personal Information was put in effect in April 2005, over-reaction and rigid compliance to this law were sometimes observed. The over-protection of personal information is sometimes criticized as an impediment to sharing information and communication. Solving these conflicts among the issues of privacy and the need to access information will be critical in the field of health informatics.

In Japan, there are several impediments to the flow and circulation of information in the fields of public health and medicine. Assessing this problem from the standpoints of creation, communication, and utilization will help to clarify each impediment to be solved. Epidemiology, which provides valid scientific evidence related to human health and health assessment, plays a crucial role in the promotion of evidence-based healthcare and the development of health informatics. It is important not only for specialists but also for the general public to acknowledge the concepts and methodologies of epidemiology. This shared understanding will further foster a system of information to facilitate appropriate decision-making that can be mutually shared among stakeholders.

The author hopes that new efforts to extend epidemiology through collaboration with individuals in other disciplines should be continued.
